# Mitochondrial disorders in children: toward development of small‐molecule treatment strategies

**DOI:** 10.15252/emmm.201506131

**Published:** 2016-03-08

**Authors:** Werner JH Koopman, Julien Beyrath, Cheuk‐Wing Fung, Saskia Koene, Richard J Rodenburg, Peter HGM Willems, Jan AM Smeitink

**Affiliations:** ^1^ Department of BiochemistryRadboud Institute for Molecular Life SciencesRadboud University Medical CenterNijmegenThe Netherlands; ^2^ Centre for Systems Biology and BioenergeticsRadboud University Medical CenterNijmegenThe Netherlands; ^3^ Khondrion BVNijmegenThe Netherlands; ^4^ Department of PediatricsRadboud Center for Mitochondrial MedicineRadboud University Medical CenterNijmegenThe Netherlands; ^5^ Department of Paediatrics and Adolescent MedicineLi Ka Shing Faculty of MedicineQueen Mary HospitalUniversity of Hong KongHong Kong

**Keywords:** children, clinical trial, drug development, mitochondria, outcome measures, Genetics, Gene Therapy & Genetic Disease, Metabolism

## Abstract

This review presents our current understanding of the pathophysiology and potential treatment strategies with respect to mitochondrial disease in children. We focus on pathologies due to mutations in nuclear DNA‐encoded structural and assembly factors of the mitochondrial oxidative phosphorylation (OXPHOS) system, with a particular emphasis on isolated mitochondrial complex I deficiency. Following a brief introduction into mitochondrial disease and OXPHOS function, an overview is provided of the diagnostic process in children with mitochondrial disorders. This includes the impact of whole‐exome sequencing and relevance of cellular complementation studies. Next, we briefly present how OXPHOS mutations can affect cellular parameters, primarily based on studies in patient‐derived fibroblasts, and how this information can be used for the rational design of small‐molecule treatment strategies. Finally, we discuss clinical trial design and provide an overview of small molecules that are currently being developed for treatment of mitochondrial disease.

**Glossary**
Blue Native‐PAGE: A polyacrylamide gel‐based electrophoresis technique that allows separation of protein complexes in their native state. It is often used for diagnostic and research purposes regarding the mitochondrial oxidative phosphorylation system.
Complementation study: A test to study and confirm the pathogenicity of a mutation based on a phenotypic screen. Often such a study is carried out by introducing the wild‐type gene in a patient (‐derived) cell line and assessing the reversal of the biological consequences of the mutated gene. The latter could involve normalization of a depolarized mitochondrial membrane potential, reversal of aberrant mitochondrial structure, or restoration of an enzymatic deficiency.
Clinical trial: A crucial part of the drug development process consisting of 4 phases. During a clinical trial (phase 1 to 3), the safety, pharmacokinetics, pharmacodynamics, and effectiveness of a compound are assessed in healthy volunteers and patient cohorts. Phase 4 consists of post‐market surveillance.
Leigh syndrome: A severe pediatric syndrome, first described by Dennis Leigh in 1951, which is caused by either mutations in mitochondrial or nuclear DNA.
LHON: Leber's hereditary optic neuropathy. A disorder that causes maternally inherited blindness. LHON is frequently caused by mutations in subunits of mitochondrial complex I, encoded by the mitochondrial DNA.
MELAS: Mitochondrial encephalomyopathy with lactic acidosis and stroke‐like episodes. A classical mitochondrial disorder primarily caused by the m.3243A>G mutation in the mitochondrial DNA.
MRI: Magnetic resonance imaging, a non‐invasive technique that uses magnetic fields and radio waves to visualize different parts of the human body. It is often applied during the diagnostic phase when a mitochondrial disorder is suspected for analysis of anatomical and physiological changes.
Outcome measures: Measures to the study the outcome of an intervention strategy such as the effect of small‐molecule treatment during a clinical trial.
Whole‐exome sequencing: A technique that allows sequencing of all protein‐coding genes within the genome. The latter is known as the exome and constitutes only a small part (∼1%) of the total genome.

## Introduction

**Figure 1:**
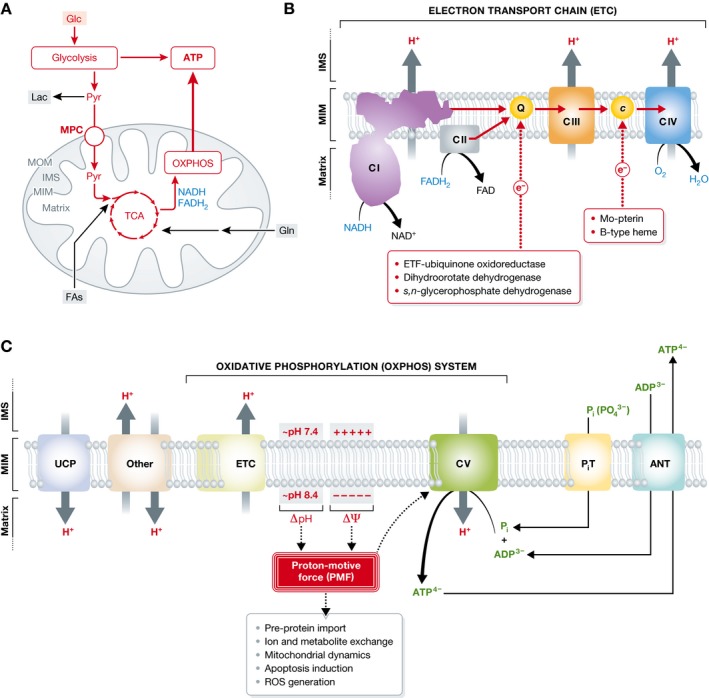
Glycolytic and mitochondrial ATP production, the electron transport chain, and oxidative phosphorylation (A) Glucose (Glc) and glutamine (Gln) enter the cell via dedicated transporters. In the cytosol, Glc is converted in the glycolysis pathway into pyruvate (Pyr), which is transported to the mitochondrial matrix by the mitochondrial Pyr carrier (MPC). Alternatively, Pyr can be converted into lactate (Lac) by the action of lactate dehydrogenase (LDH). Inside the mitochondrial matrix, Pyr is converted in to acetyl coenzyme A (acetyl‐CoA; not shown) by pyruvate dehydrogenase (PDH) to enter the tricarboxylic acid (TCA) cycle. The latter supplies the oxidative phosphorylation system (OXPHOS) with substrates in the form of reduced nicotinamide adenine dinucleotide (NADH) and reduced flavin adenine dinucleotide (FADH_2_). In addition, Gln can enter the mitochondrial matrix where it is converted by glutaminase into glutamate (not shown), a TCA cycle substrate. Also fatty acids can enter the mitochondrial matrix and enter the TCA cycle following their conversion into acetyl‐CoA (not shown). In healthy cells, the conversion of Glc into Pyr and its further metabolic conversion by the TCA and OXPHOS system constitute the major pathway for ATP generation (marked in red). (B) The mitochondrial electron transport chain (ETC) consists of 4 multisubunit protein complexes (complex I to IV) that are embedded in the mitochondrial inner membrane (MIM). Electrons are donated by NADH (at complex I) and FADH_2_ (at complex II) to coenzyme Q_10_ (Q), which transports them to complex III. From thereon, electrons are transported to complex IV by cytochrome *c* (*c*) where they are donated to molecular oxygen (O_2_). Although not discussed here, in addition to the ETC complexes also other proteins can provide coenzyme Q_10_ and cytochrome *c* with electrons (red boxes) in a tissue‐dependent manner. During electron transport, energy is liberated and used expel protons (H^+^) from the mitochondrial matrix intro the inter‐membrane space (IMS) between the MIM and mitochondrial outer membrane (MOM). As a consequence, the mitochondrial matrix displays an increased pH and the MIM has a highly negative‐inside membrane potential (Δψ). (C) Together, the pH (ΔpH) and potential difference (Δψ) across the MIM determine the magnitude of the proton‐motive force (PMF), which is used by the F_o_F_1_‐ATPase (complex V) to drive mitochondrial ATP production from inorganic phosphate (P_i_) and ADP. In addition to ATP generation, virtually all other mitochondrial processes including ion exchange and pre‐protein import require a proper ΔpH and/or Δψ. The magnitude of the PMF not only depends on the combined action of the ETC and complex V (i.e., the oxidative phosphorylation system; OXPHOS) but also is affected by other electrogenic systems. These include uncoupling proteins (UCPs), the P_i_ transporter (P_i_T), and the adenine nucleotide translocator (ANT). This figure was compiled based on (Koopman *et al*, 2010; Valsecchi *et al*, 2010; Liemburg‐Apers *et al*, 2011; Koopman *et al*, 2012, 2013 and Liemburg‐Apers *et al*, 2015).

Mitochondria are semi‐autonomous organelles that are present in the cytosol of virtually all cells. They consist of a double‐membrane system that envelops the mitochondrial matrix compartment. Functionally, mitochondria are key players in cellular ATP production, fatty acid oxidation, heme biosynthesis, apoptosis induction, heat generation, and calcium homeostasis. Mitochondrial diseases are an expanding group of disorders of which the first signs and symptoms can become apparent from prenatal development to late adulthood (Koopman *et al*, 2012, 2013; Vafai & Mootha, 2012; Chinnery, 2015; Lightowlers *et al*, 2015). These disorders can be defined as “primary” (i.e., arising from a mutation in one of the genes encoding a mitochondria‐localized protein) or “secondary” (i.e., arising from an external influence on mitochondria). The latter include off‐target effects of cholesterol‐lowering statin drugs (e.g., Schirris *et al*, 2015) and defects in the normal breakdown of branched‐chain amino acids as in propionic acidurias, which cause severe combined respiratory chain deficiencies (e.g., Schwab *et al*, 2006). Here, we primarily focus on mitochondrial diseases that (i) clinically manifest themselves before the age of 18 years and (ii) arise from mutations in nuclear DNA (nDNA)‐encoded structural proteins or assembly factors of the mitochondrial oxidative phosphorylation (OXPHOS) system. The mitochondrial OXPHOS system is embedded in the mitochondrial inner membrane (MIM) and represents the final step in the conversion of nutrients to energy by catalyzing the generation of ATP (Fig 1A). This process is carried out by the combined action of the mitochondrial electron transport chain (ETC) complexes (Fig 1B) and the ATP‐producing F_o_F_1_‐ATPase (complex V; Fig 1C). The ETC consists of four multiprotein complexes (complex I to complex IV). Complex I and complex II abstract electrons from reduced nicotinamide adenine dinucleotide (NADH) and reduced flavin adenine dinucleotide (FADH_2_), respectively. Subsequently, these electrons are donated to the electron carrier coenzyme Q_10_, which transports them to complex III. From thereon, electrons are transferred to the electron carrier cytochrome *c* and transported to complex IV. At the latter complex, the electrons are donated to molecular oxygen (O_2_) to form water. The energy released by the electron transport is used to drive trans‐MIM proton (H^+^) efflux from the mitochondrial matrix by complexes I, III, and IV, thereby creating an inward‐directed proton‐motive force (PMF). The latter consists of a chemical (ΔpH) and an electrical component (Δψ; Fig 1C), and is used by complex V to generate ATP by chemiosmotic coupling (Mitchell, 1961). In addition, ΔpH and/or Δψ are essential in sustaining virtually all other mitochondrial functions like the import of pre‐proteins from the cytosol and ion/metabolite exchange (Fig 1C). In normal healthy cells (Fig 1A; red), cellular ATP is predominantly generated through subsequent metabolic reactions of the glycolysis pathway (cytosol), the pyruvate dehydrogenase complex (PDHC, mitochondrial matrix), the tricarboxylic acid (TCA) cycle (mitochondrial matrix), and the OXPHOS system (MIM). This ATP is used to fuel energy‐consuming cellular processes.

## Diagnosing OXPHOS disease in children

**Figure 2:**
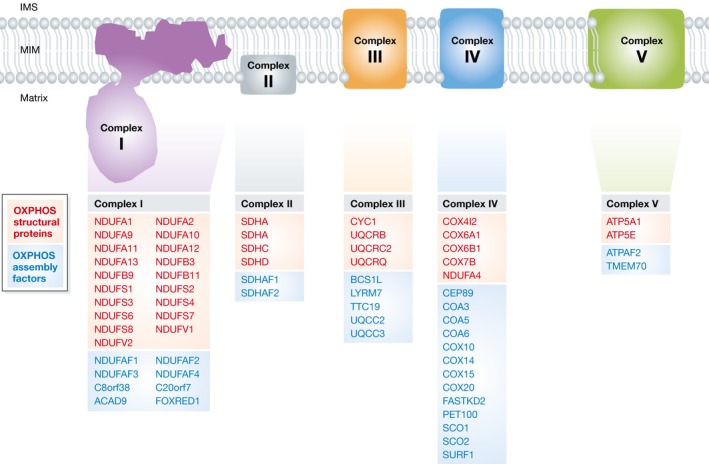
Currently identified mutations in nDNA‐encoded OXPHOS subunits and assembly factors causing mitochondrial disease in children OXPHOS structural subunits in which a pathological mutation was reported are depicted in red for each complex. Assembly factors are indicated (in blue). The data in this figure were compiled from (Koopman *et al*, 2012, 2013; Mayr *et al*, 2015; Ostergaard *et al*, 2015; van Rahden *et al*, 2015).

The five complexes of the mitochondrial OXPHOS system are assembled from 92 distinct proteins, requiring the assistance of at least 37 nDNA‐encoded assembly factors (Nouws *et al*, 2012; Koopman *et al*, 2013). Each OXPHOS complex except complex II is of bi‐genomic origin and contains subunits encoded by mitochondrial DNA (mtDNA) and nDNA (Koopman *et al*, 2013). Complex I is built up from 44 subunits (7 mtDNA‐ and 37 nDNA‐encoded), complex II of 4 subunits, complex III of 11 subunits (1 mtDNA, 10 nDNA), complex IV of 14 subunits (3 mtDNA, 11 nDNA), and complex V of 19 subunits (2 mtDNA, 17 nDNA). As mentioned in the introduction, we here primarily focus on mitochondrial diseases arising from mutations in nDNA‐encoded structural OXPHOS subunits and assembly factors (Fig 2). Since the first description by Luft of a patient with exercise intolerance and hyperthermia (Luft *et al*, 1962), the clinical spectrum of diseases associated with OXPHOS malfunction has tremendously broadened. In our opinion, a mitochondrial disease should be considered in any patient presenting with an unexplained mono‐ or multisystemic disease, whether progressive or not. Applying this relatively broad definition has important consequences for the diagnostic approach in children, which is truly challenging given the general lack of awareness within the broader medical community, the relatively low incidence of mitochondrial disease, and the complex genotype–phenotype relationship typical of these inborn errors (Smeitink, 2003). It is also important to increase awareness for mitochondrial disorders to shorten the potentially long delays between appearance of the first symptoms and diagnostic analysis and confirmation of the defect. Regarding the various clinical presentations of mitochondrial OXPHOS disease, detailed reviews have been presented elsewhere (Kisler *et al*, 2010; Koene & Smeitink, 2011; Lake *et al*, 2016). The majority of children suffering from mitochondrial OXPHOS disease lack a specific syndromic appearance and some do not display increased blood lactate levels (Triepels *et al*, 1999). In case of neurodegenerative diseases, mutations in OXPHOS subunits (mtDNA‐ or nDNA‐encoded) and assembly factors have, for instance, been associated with (Koopman *et al*, 2013): Leigh (‐like) syndrome, leukoencephalopathy, MELAS (mitochondrial encephalomyopathy, lactic acidosis, and stroke‐like episodes) syndrome, NARP (neuropathy, ataxia, and retinitis pigmentosa), Parkinsonism/MELAS, and (susceptibility) modification of PD (Parkinson disease), and AD (Alzheimer's disease).

### The diagnostic process

When clinical signs and symptoms suggest a mitochondrial disorder, the following general strategy is carried out: (i) a detailed analysis of the medical/family history to discriminate between maternal (mtDNA) and Mendelian (nDNA) inheritance, (ii) evaluation of mitochondrial biomarkers like lactate, pyruvate, alanine, fibroblast growth factor 21 (FGF21; Suomalainen *et al*, 2011) and growth and differentiation factor 15 in blood (GDF15; Yatsuga *et al*, 2015) or the excretion of TCA cycle intermediates in urine, and (iii) detailed investigations of affected organs and tissues by medical specialists like neurologists, ophthalmologists, cardiologists, and/or by imaging techniques such as echocardiography or magnetic resonance imaging (MRI) of brain, heart, and skeletal muscle, and by neurophysiological studies (such as obtaining an electroencephalogram; EEG). The decision to perform a skeletal muscle biopsy for histopathological, immunohistochemical, and/or enzymological analysis is taken based on the “Bernier” or “Wolf/Smeitink” mitochondrial disease criteria (MDC; Bernier *et al*, 2002; Wolf & Smeitink, 2002), which might include biochemical (e.g., ATP production rate) and general criteria (e.g., clinical presentation) to establish the probability of a mitochondrial disease. Biochemical analysis usually includes spectrophotometric activity analysis of individual OXPHOS complexes. Blue Native‐PAGE, which can be used to assess the relative amount of fully assembled OXPHOS complexes, can also be a useful tool (e.g., van Rahden *et al*, 2015). In addition to muscle tissue, enzymes are often examined in cultured skin fibroblasts. In combination with a muscle biopsy, both positive and negative fibroblast results are informative with respect to predicting the underlying genetic defect. Genetic analysis can be carried out either by traditional candidate gene sequencing, or by more comprehensive approaches including direct (whole) exome sequencing (in which all coding regions in the genome are sequenced) or whole‐genome sequencing, often followed by functional complementation analysis in patient‐derived cells.

### The impact of whole‐exome sequencing

The implementation of whole‐exome sequencing as a routine molecular diagnostic test for mitochondrial disorders has resulted in the identification of numerous variants in many different genes (DaRe *et al*, 2013; Lieber *et al*, 2013; Ohtake *et al*, 2014; Taylor *et al*, 2014; Wortmann *et al*, 2015). Genetic variants include known pathogenic mutations, the clinical/biochemical phenotype of which matches those described in the literature and public databases. In this case, the diagnosis is clear and no further functional tests are necessary. When an unknown gene variant is encountered, there are several possibilities: (i) the variant occurs in a gene that is associated with a known disease and interferes with the function of the gene product; again, this means that no additional functional tests are necessary, (ii) the variant results in an amino acid change at the protein level or possibly interferes with the expression of a disease‐associated gene and/or the splicing of its mRNA; this means that the consequences of the variant are not unambiguously predictable and a functional test can help to establish the diagnosis, (iii) the variant occurs in a gene of unknown function or a gene not previously associated with disease; this means that the diagnosis requires a functional confirmation of the defect. In general, mitochondrial disorders display a poor correlation between genotype and phenotype. This further emphasizes the importance of functional validation of genetic variants (Daud *et al*, 2015). For genes known to be mutated in OXPHOS disease, the possibility to perform relatively low‐cost diagnostic exome sequencing using DNA extracted from blood samples has certainly sped up and facilitated the diagnostic process (Wortmann *et al*, 2015). However, it is important to realize that when a negative result is obtained, this does not conclusively rule out the presence of a mitochondrial disease. This is caused by the fact that: (i) the exome sequencing technique displays non‐optimal coverage meaning that mutations in non‐covered parts of the exome cannot be excluded, (ii) even a whole exome covers only approximately 1% of the whole genome, (iii) the pathophysiological effects of many polymorphisms are unknown, and (iv) the number of mutated genes demonstrated to be involved in mitochondrial disease is continuously increasing. As an example of the latter, we demonstrated that a patient with intellectual disability and multisystemic problems displayed an isolated enzymatic complex IV deficiency caused by a pathological mutation in the centrosomal protein of 89 kDa (CEP89), previously not associated with mitochondrial dysfunction (van Bon *et al*, 2013). As a consequence, it becomes increasingly difficult to classify mitochondrial diseases in children based upon their phenotypic presentation. Therefore, the number of patients that will be correctly diagnosed with a mitochondrial (OXPHOS) disease will be underestimated. A possible solution to this problem could be that patients for whom an obvious explanation for their symptomatology cannot be established are referred to specialized (inter)national centers of excellence (e.g., the Radboud Center for Mitochondrial Medicine in the Netherlands), which can decide on an appropriate cost‐ and time‐effective diagnostic strategy.

### The relevance of complementation studies

Genetic complementation studies are carried out by introducing the wild‐type DNA of the mutated gene into patient‐derived cells (usually skin fibroblasts). DNA transduction by lentiviral transmission is an efficient way to achieve genetic complementation (e.g., Jonckheere *et al*, 2011). Although it is relatively time‐consuming, this procedure yields a population of stably complemented cells. Transient transfections can also be used, for instance, using an insect virus (baculovirus) system (e.g., Kirby *et al*, 2004; Hoefs *et al*, 2008). In this approach, the protein to‐be‐overexpressed can be tagged with a fluorescent protein (e.g., Hoefs *et al*, 2008), or be part of an IRES (internal ribosome entry site)‐containing construct (e.g., Bouabe *et al*, 2008). Using the IRES strategy allows simultaneous expression of the protein and a fluorescent marker protein without them being physically attached to each other. Although with a transient transfection protocol, not all cells are necessarily transfected, it can be advantageous when a microscopic readout is used since it allows side‐by‐side comparison of transfected (complemented) and non‐transfected (non‐complemented) patient cells. Depending on the nature of the mutation, functional confirmation of pathogenicity can obtained using a relatively simple readout like enzymatic activity (Haack *et al*, 2012; Ngu *et al*, 2012; Jonckheere *et al*, 2013; Szklarczyk *et al*, 2013) or protein expression levels (assessed by Western blotting; Hoefs *et al*, 2008). More advanced strategies include comparative analysis of control, patient, and complemented patient primary fibroblasts with respect to mitochondrial morphology (Koopman *et al*, 2005; Jonckheere *et al*, 2011) and Δψ (Hoefs *et al*, 2008). Unfortunately, functional studies of the genetic defect are often lacking in published studies, especially when a large number of patients have been analyzed. Although large‐scale complementation and other functional studies may be practically challenging, they should be carried out to prevent detected variants prematurely or erroneously ending up in pathogenic mutation databases. For example, a recent study described two novel mutations in SCO2 (a complex IV assembly gene) in patients with early‐onset myopia (Jiang *et al*, 2014). Although the authors did state that additional studies are needed to prove the pathogenicity of these (and other) mutations, the latter are nevertheless listed in the Human Gene Mutation Database (HGMD) as “disease‐causing” ( www.hgmd.cf.ac.uk). This is not to criticize mutation databases as these are extremely valuable to clinical practice, but to illustrate that clinicians and laboratory specialists should remain critical about the information provided by these databases. Similarly, in publications dealing with genetic variants, incorrect or incomplete information can have important clinical implications for individual patients.

## Cellular pathophysiology of OXPHOS dysfunction

During the last 15 years, we have strongly advocated the use of living cells (in addition to cell homogenates, isolated mitochondria, and fixed tissue samples), to study the pathophysiology of mitochondrial dysfunction in general and OXPHOS mutations in particular (e.g., Smeitink *et al*, 2004; Koopman *et al*, 2010, 2012, 2013; Willems *et al*, 2015). Live‐cell analysis is crucial since mitochondrial and cellular functions are tightly interconnected. This is illustrated by the following observations: (i) mitochondrial morphology and function are altered by isolation procedures, (ii) the cellular environment is required to provide substrates to mitochondria and the OXPHOS system, (iii) mitochondria exchange ions and metabolites (e.g., ADP, P_i_ and ATP) with the cytosol, and (iv) mitochondrial and cellular functioning are linked and controlled by (retrograde) signaling pathways (e.g., Palmieri, 2008; Koopman *et al*, 2010; Picard *et al*, 2011; Szabo & Zoratti, 2014). In principle, a mutation in an OXPHOS protein‐encoding gene can reduce the catalytic activity of the OXPHOS complex, its protein levels, or both. In case of isolated complex I deficiency, the reduction in the levels of fully assembled complex I in primary patient fibroblasts was linked to a proportional decrease in complex I residual activity (e.g., Valsecchi *et al*, 2010). This suggests that mutations in nDNA‐encoded complex I subunits reduce the biosynthesis of fully active complex I and/or induce complex I destabilization (i.e., an “expression defect”). Only in a single case (i.e., a patient carrying an Asp446Asn mutation in the *NDUFS2* gene of complex I), a reduced complex I activity was not paralleled by a lower level of fully assembled complex I (i.e., a “catalytic defect”; Ngu *et al*, 2012). Quantitative live‐cell microscopy of primary fibroblasts from 14 control subjects and 24 children with isolated complex I deficiency revealed that patient cells displayed a less negative Δψ (i.e., “depolarization”), increased mitochondrial NADH levels, elevated reactive oxygen (ROS) levels, aberrations in mitochondrial morphology, and disturbed cytosolic and mitochondrial Ca^2+^ and ATP handling (Koopman *et al*, 2005, 2007, 2008; Verkaart *et al*, 2007a; Golubitzky *et al*, 2011; Valsecchi *et al*, 2010; Roestenberg *et al*, 2012; Leman *et al*, 2015). Interestingly, several of the above aberrations were also observed in fibroblasts carrying mutations in other OXPHOS complexes and assembly factors (e.g., Szklarczyk *et al*, 2013), in mouse cells with genetic complex I deficiency (e.g., Kruse *et al*, 2008; Valsecchi *et al*, 2012, 2013), in cells treated with OXPHOS inhibitors (e.g., Forkink *et al*, 2014, 2015; Distelmaier *et al*, 2015; Schöckel *et al*, 2015), and in cells with mutations in non‐OXPHOS mitochondrial proteins (e.g., Mortiboys *et al*, 2008; Heeman *et al*, 2011; Hoffmann *et al*, 2012). This suggests that, depending on the cellular context and experimental conditions, the observed aberrations in primary skin fibroblasts of patients with inherited complex I deficiency reflect the “general” consequences typical of mitochondrial dysfunction. Correlation analysis suggested that NADH and ROS levels increased as a direct consequence of the reduced expression of fully active complex I (Verkaart *et al*, 2007a,b). Moreover, patient fibroblasts with a moderate reduction in complex I activity displayed a much smaller increase in ROS levels than fibroblasts with a large reduction in complex I activity (Koopman *et al*, 2007). In addition, mitochondrial morphology was not affected and appeared fragmented when complex I activity was moderately and greatly reduced, respectively (Koopman *et al*, 2007). Integrated analysis of cellular and clinical data revealed that fibroblasts from patients with a relatively early age of disease onset/death displayed a large reduction in complex I activity. Similarly, patients with a later age of disease onset/death displayed a much smaller reduction in complex I activity (Blanchet *et al*, 2011). The same study revealed that the extent of complex I deficiency was proportional to the cellular level of OXPHOS proteins and inversely related to cellular NADH/ROS levels and degree of mitochondrial fragmentation.

Taken together, the above results suggest that complex I activity in patient fibroblasts might be improved by reducing the increased ROS levels and normalizing mitochondrial morphology. Indeed, treating patient fibroblasts with the water‐soluble vitamin E‐derived antioxidant Trolox (6‐hydroxy‐2,5,7,8‐tetramethylchroman‐2‐carboxylic acid) reduced ROS levels and increased the expression of fully assembled complex I and thereby complex I activity (Koopman *et al*, 2008). A follow‐up study demonstrated that Trolox treatment reversed Δψ depolarization and normalized Ca^2+^‐stimulated ATP production in patient fibroblasts (Distelmaier *et al*, 2009). Further mechanistic analysis revealed that ROS and thiol redox state co‐control mitochondrial morphology and function in human skin fibroblasts (Distelmaier *et al*, 2012; Willems *et al*, 2013), compatible with the apparent link between ROS levels and regulation of mitochondrial morphology and function (Willems *et al*, 2015). This suggests that the reduced complex I activity, increased ROS level, and altered mitochondrial morphology, and possibly also other aberrations, might constitute linked therapeutic targets (Koopman *et al*, 2007; Blanchet *et al*, 2011, 2015). We thus pursued antioxidant treatment as a potential strategy to mitigate the cellular aberrations in fibroblasts from patients with isolated complex I deficiency. This is discussed further in the section on the development of the novel antioxidant KH176 (see below).

## Development of treatments

### Overall strategy

A “lead compound” is a chemical molecule displaying pharmacological or biological activity likely to be therapeutically useful, but with a suboptimal chemical structure that requires modification to acquire better drug‐like properties. Such properties would include efficacy, potency, safety, synthesis route, cost, chemical stability, metabolic stability, and, last but not least, patentability (Pritchard *et al*, 2003). During optimization, the primary selection criterion is usually based on compound efficacy and potency. However, compounds active in one assay may perform poorly in others, indicating that their selection needs to be based on multiple properties. Lead optimization and selection mainly depend on the properties of the molecule in studies involving its absorption, distribution, metabolism, and excretion (ADME properties), its pharmacokinetics (PK) profile, and *in vitro* safety. Unfortunately, there are no predictive rules for identifying a potentially successful compound. During the selection process, certain additional constraints can be applied depending on the desired *in vivo* properties of the small molecule. These include the preferred route of administration (e.g., oral bioavailability), the targeted organs (e.g., blood–brain permeability), or potential drug–drug interactions in the studied disease group. Importantly, a certain amount of specific preclinical information is necessary to fulfill the requirements of the ethical/regulatory authorities involved and allow proceeding to the next development phase. To illustrate this process, we here present some of our own experiences in further developing an active pharmaceutical ingredient (API) following lead optimization. This consists of the following steps (Table 1):

**Table 1:** Steps in the development of active pharmaceutical ingredients (APIs) toward clinical use

Phase	Subphase	Activities	Outcomes
Lead optimization (non‐GLP)	*In vitro* ADME	Chemical stability, solubility, metabolic stability, stability in gastric environment, CYPs interaction, PPB, BBB permeability	Information required for the final selection of the lead compound; it is usually performed on a group of the most potent hit analogs developed during the hit‐to‐lead screen
	*In vitro* toxicology	Cytotoxicity, hepatotoxicity, cardiotoxicity, mutagenicity	
	*In vivo* ADME	Pharmacokinetics: (oral) bioavailability, plasma exposure, tissue distribution, metabolism, excretion	
Metabolite profiling	N.a.	Profiling and identification of metabolites formed in hepatocytes of humans and animal species used for non‐clinical safety	Allows the selection of animal species for further *in vivo* toxicology studies
Synthesis scale‐up (GMP)	N.a.	Synthesis of a large batch of API in a GMP fashion	A GMP batch of API produced in quantity sufficient to cover the toxicology and First‐in‐Human studies
CMC of the API	N.a.	Validation of API chemical structure; determination of physicochemical parameters	Mandatory information for IB/IMPD or IND filling and for formulation development
API stability	N.a.	Stability study of the API under ICH conditions	Expiry date of the API in different storage conditions
Pre‐formulation and stability of drug product	N.a.	Selection of the formulation based on physicochemical properties of the API and desired route of administration; determining API stability in the formulation	Optimal formulation and stability data to support GLP toxicology and First‐in‐Human studies
GLP‐compliant API detection methods	N.a.	Validation of a GLP‐compliant method to quantify the API in dose formulation studies and biological fluids	GLP‐compliant and validated method to detect and quantify API during toxicology and First‐in‐Human trials
Non‐clinical safety	Pilot toxicology study	Dose range finding and 14‐day toxicology study in a single animal species	First information on the dose range and specific toxicity to be monitored during the next phases
	GLP‐compliant regulatory toxicology study	*In vitro*: GLP‐compliant *in vitro* toxicology study	Genetic toxicology, safety pharmacology
		*In vivo*: GLP‐compliant *in vivo* toxicology	General toxicology
		*In vivo*: reproductive toxicology study	Adverse effects on embryonic development
		*In vivo*: juvenile toxicology study	Specific adverse effect on juvenile population
Drug–drug interaction study	N.a.	Inhibition and substrate potential of API toward CYPs, drug transporters, and UGTs	Prediction of potential adverse effect arising from polypharmacology (i.e., how others drugs and API will impact on each other's ADME properties leading to modified PK/PD profile)
Investigational brochure (IB)	N.a.	Compile all preclinical data relevant to study the API in humans under ICH conditions	The IB is required for the clinical investigator to design the clinical trial protocol
Investigational medical product dossier (IMPD)	N.a.	Complete the IMPD form with CMC information about drug substance and drug product	The IMPD is required for authorization to enter First‐in‐Human trials
Clinical trial application (CTA) form	N.a.	Complete CTA with information about drug product and trial design, site and investigator	The CTA is required to obtain authorization to enter First‐in‐Human trials

This table summarizes the overall preclinical experimental and administrative steps required for a novel API to entering First‐in‐Human trials. It is based upon experiences within our SME Khondrion and is not intended to be exhaustive. Each API will have a specific development path, and API developers should strictly follow the guidance and requirements from the official agencies involved.

ADME, absorption, distribution, metabolism, and excretion; API, active pharmacological ingredient; BBB, blood–brain barrier; CMC, chemistry and manufacturing controls; CTA, clinical trial application; GLP, good laboratory practices; GMP, good manufacturing practices; CYP, cytochrome P450; IB, investigational brochure; ICH, The International Conference on Harmonisation (of Technical Requirements for Registration of Pharmaceuticals for Human Use); IMPD, investigational medical product dossier; IND, investigational new drug; N.a., not appropriate; PD, pharmacodynamics; PK, pharmacokinetics; PPB, plasma protein binding; UGT, UDP glucuronosyltransferase.

- Metabolic profiling in hepatocytes of humans and various animal species will assist in deciding which animal species to use during the regulatory toxicology phase, in which the API's toxic potential is assessed. As any metabolite can cause toxicity, the combined metabolites detected in two selected animal species should cover all potential metabolites in humans.
- Scaling up of API synthesis according to GMP (good manufacturing practice) standards, acquiring CMC (chemistry and manufacturing controls) information of the API, and determining API stability. Information obtained during these three steps is crucial for defining the formulation of the API and obtain authorization to enter the clinical trial phase. Producing a GMP batch of the API is not mandatory for the non‐clinical toxicology phase but it is strongly advised to use the same GMP batch for both regulatory toxicology studies and the early trial clinical phases. This relates to the fact that it needs to be demonstrated that various GMP batches display an identical (im)purity profile.
- Optimization of a good laboratory practice (GLP)‐compliant method of detection of the API in blood or plasma in order to study its pharmaco‐ and toxicokinetic profile during non‐clinical toxicology and clinical trials.
- Performing a non‐clinical regulatory toxicology study. This study will be designed based on specific toxicity information obtained during *in vitro* or *in vivo* screening and the intended patient populations (e.g., reproductive toxicology or juvenile toxicology). The duration of this study will depend on the expected duration of the treatment in patients during clinical trials and will help to determine the safe starting dose for administration to humans.
- Investigating drug–drug interactions (DDI). The different medicine authorities have issued strict guidelines regarding these studies. Although not mandatory for clinical phase 1 studies (healthy volunteers), DDI studies are of crucial importance prior to executing patient trials (phase 2 clinical trials and beyond). This is to avoid potential API toxicity due to interaction with medications or deficiencies in liver or kidney metabolism.
- Compiling the investigational brochure (IB). This contains all preclinical (pharmacology and toxicology) data relevant for use of the API in the clinic. The IB will be continuously updated during clinical development.
- Compiling the investigational medical product dossier (IMPD). This dossier contains all physicochemical and drug formulation information of the API.
- Completing the clinical trial application (CTA) form. The CTA contains information about the drug product, trial design, and investigator details. Together with the IMPD, the CTA is required for approval of the clinical trials.

### Designing clinical trials

Before an API obtains market approval and can therefore be prescribed by clinicians, in‐human studies have to be performed. Classically, these studies consist of four phases. In phase 1, API pharmacokinetics and dynamics (including toxicity) are determined in healthy volunteers. During phase 2, the intention is to determine the efficacy of the API in a small and homogeneous patient group. Phase 3 seeks to confirm the effectiveness and safety of the API on clinically relevant outcome measures in a large and more heterogeneous group of patients. The results of phase 3 are mostly used to apply for market authorization. Following market authorization, the (long‐term) safety and efficacy of the drug remain to be monitored (phase 4). One of the most challenging aspects of clinical trials involves the selection, validation, and application of clinically relevant outcome measures.

### Outcome measures

Outcome measures provide information about certain disease aspects, such as severity, response to therapy or remission (Brunner & Ravelli, 2009). In case of frequently occurring disease (e.g., cardiovascular disease, cancer), the time‐to‐event (relapse, stroke, myocardial infarction, death), histology, or radiological results are often used as outcome measures (Preiss *et al*, 2011; Fernandez‐Martos *et al*, 2012; Etzioni *et al*, 2015). Obviously, the credibility of a clinical trial greatly depends on the chosen outcome measures and the strategy that is used to quantify them (Tugwell & Boers, 1993). In general, the number of patients required for a clinical trial (sample size) depends on the magnitude of the expected effect relative to the precision of the used measurement. This means that either the treatment should produce a large effect or the used outcome measures should be very precise when only a small patient cohort is available, as is the case for children with OXPHOS disease. Moreover, if the therapy under development slows down disease progression rather than reducing morbidity (Friedman *et al*, 2010; Kang, 2013), it becomes challenging to identify outcome measures suited for quantitative analysis. In addition, children are continuously growing and developing (meaning that age‐specific tests and age‐ and size‐matched reference values are required) and their understanding and enthusiasm, as well as their cooperation, can affect the test results. Since virtually all the functional tests require the child to perform at his/her maximal level, children with mild mental disability might not give consistent results because of attention deficits and a lack of understanding the importance of the tests. Measuring disease progression in children that are severely mentally disabled and sometimes not able to communicate is virtually impossible. In general, the outcome measures not only depend on the disease stage or disability grade but also on age, gender, intellectual abilities, environment, and personal factors. To the best of our knowledge, there are no established outcome measures that correlate with disease progression in mitochondrial dysfunction (Lee *et al*, 2010; Augustine *et al*, 2013; Koene *et al*, 2014, 2015). In case of mitochondrial OXPHOS disorders with neuromuscular dysfunction, the regulatory authorities [i.e., the European Medicines Agency (EMA) and the American Food and Drug Adminstration (FDA)] advise using the 6‐min walking test (6MWT) as a primary outcome measure. This test measures the total distance an individual is able to walk during 6 min on a hard and flat surface (Balke, 1963). In other disease fields (e.g., Dubrovsky *et al*, 2013; Alfano *et al*, 2014), as well as in our own experience with OXPHOS disease patients (Dirks *et al*, 2015), the 6MWT showed a considerable test–retest variability and lack of sensitivity. Therefore, we expect that other approaches like accelerometry, continuously measuring the physical activity of the child in their home environment, or measuring detailed gait characteristics are more appropriate to detect subtle but clinically relevant changes in disease severity (S. Koene *et al*, unpublished findings). In addition we have selected several clinically relevant and robust outcome measures to be used in clinical trials with children suffering from mitochondrial disease. These outcome measures were chosen based upon the most burdensome complaints/symptoms according to the patients and their parents (Koene *et al*, 2013a), and on the results of validation studies in Duchenne muscular dystrophy and cerebral palsy, which were used as a model for mitochondrial myopathy and mitochondrial encephalopathy, respectively. The total set of outcome measures can be divided into a “common core set” including the following: accelerometer test, 6MWT test, Gross Motor Function Measure (GMFM) and Medical Research Council (MRC) scale; and a number of “optional tests” including: forced vital capacity, Child Health Questionnaire, and growth charts (Koene *et al*, 2013b). The validity of these outcome measures was assessed in several studies (Dirks *et al*, 2015; S. Koene *et al*, unpublished findings), which suggested that accelerometry and 2‐dimensional (2D) speckle tracking echocardiography (Blessberger & Binder, 2010) are feasible instruments to monitor disease progression, but the assisted 6‐minute cycling test is not. The currently available diagnostic biomarkers for mitochondrial disease (i.e., GDF15, FGF21) do correlate with disease severity. However, these biomarkers appear not to be suited for monitoring disease progression since the change in the concentration of neither biomarker correlated to the change in disease severity (Koene *et al*, 2014, 2015). Additional validation studies are required to guide the further selection of outcome measure(s) for use in clinical trials involving children. Obviously, this selection is highly dependent on the mode of action of the API being tested and is preferably made by integrating the expertise of pediatricians and other medical specialists, statisticians, regulatory experts, industry partners, and patient/parent representatives.

### Study population

In case of mitochondrial OXPHOS diseases, only a low number of patients are available for phase 2 clinical trials. This means that low patient‐to‐patient variation is required to demonstrate the potential efficacy of a small molecule. In the ideal case, the used patient cohort should display a homogeneous genotype, phenotype, disease stage, and organ specificity. Preferentially, the patients should neither be on medication nor display liver or kidney failure (Pfeffer *et al*, 2013). However, mitochondrial OXPHOS syndromes with a relatively high level of homogeneity for which a reasonable number of patients are available (e.g., Leber's hereditary optic neuropathy; LHON) are uncommon and hardly any of them is pediatric. Given this fact, the difficulty of assessing outcome measurements in children (see above), and ethical and regulatory considerations, phase 2 clinical trials should preferentially be carried out first in adult mitochondrial disease patients. In addition to mitochondrial syndromes caused by OXPHOS mutations, also other rare pediatric syndromes exist. An example of such a disease is Barth syndrome, caused by a mutation in the *TAZ* gene. The latter encodes the tafazzin protein, which is involved in the synthesis of the mitochondrial lipid cardiolipin. Clinically, Barth syndrome appears to display a reasonably homogeneous phenotype characterized by skeletal muscle weakness, cardiomyopathy, neutropenia, and growth delay (Barth *et al*, 1983; Spencer *et al*, 2006; Roberts *et al*, 2012; Jefferies, 2013; Rigaud *et al*, 2013). This suggests that a Barth syndrome patient cohort might be suited for phase 2 clinical trials. On the other hand, because OXPHOS dysfunction in Barth syndrome is a consequence of mitochondrial membrane alterations, such a cohort might not be the best choice for drug efficacy analysis since the obtained results might not be generalizable to primary OXPHOS disorders. Alternatively, Kearns Sayre syndrome is another more or less homogeneous mitochondrial disease that is characterized by ophthalmoparesis, pigmentary retinopathy, deafness, muscle weakness, ataxia, and cardiac conduction block (Yamashita *et al*, 2008). Although not strictly pediatric (i.e., it can manifest itself in children and adolescents), the progression of Kearns Sayre syndrome appears to be reasonably stable (Grady *et al*, 2014). Since most Kearns Sayre syndrome patients are mentally competent, the feasibility and ethical acceptability of clinical trial is increased. However, separate studies will have to be performed in neonates, infants, and young children. Another strategy is to include all children diagnosed with a mitochondrial disorder, regardless of their genetic or biochemical diagnosis, using very strict clinical inclusion criteria. The latter can include that the child: (i) has a stable disease course in the past year, (ii) can follow instructions such as hopping on one leg or rotating a pen in the hand, (iii) scores a 6MWT value between the age‐ and height‐matched 5^th^ and 10^th^ percentile, and/or (iv) a GMFM score between 15 and 25%. Such an approach requires a separate neonatal study, including for example neonates with infantile mitochondrial lactic acidosis, since the pharmacokinetics, safety, and efficacy in neonates might differ from the other (pediatric) groups. For all phase 2 trials, it is crucial to realize that children are clinically and physiologically not equivalent to “small adults”. This means that designing a proper drug administration regime requires separate studies on drug formulation, safety, and pharmacokinetics.

### Study design

In case of pediatric OXPHOS disease, it is difficult, if not impossible, to carry out classical randomized control clinical trials since these require relatively large homogeneous patient cohorts. However, alternative trial designs are applicable (Chow & Chang, 2008; Gupta *et al*, 2009; Cornu *et al*, 2013). In the “crossover” and “*n*‐of‐1” designs, each patient serves as its own control and receives both placebo and active drug treatment, which facilitates recruitment. Whereas a crossover study aims to determine the efficacy of a drug in a group of patients, an *n*‐of‐1 study aims to determine drug effects within a single subject, although the results of multiple *n*‐of‐1 studies can be used for meta‐analysis (Gupta *et al*, 2009). Formally, crossover and *n*‐of‐1 designs can only be applied if the disease status is similar between the start of the first and the second treatment phase (i.e., similar baseline measurements before the placebo or active drug phase). In this sense, they are less compatible with pediatric mitochondrial OXPHOS disorders. However, if the duration of the drug treatment and the washout period (being the time between the treatment phases in which the drug and the long‐term drug effects are eliminated from the body before the start of the second phase) is short, and no long‐term effects of treatment are expected (i.e., minimal carryover effect), these designs may be of great value when evaluating drug efficacy in mitochondrial disease. If feasible, larger and phenotypically homogeneous cohorts can be analyzed in crossover studies. In ultra‐rare mitochondrial syndromes or those with a heterogeneous phenotype, *n*‐of‐1 trials are most suitable. In both cases, patients with an obviously oscillating disease course should be excluded. When choosing a crossover or *n*‐of‐1 design, several other methodological issues should be covered. First of all, the sequence of the placebo and the active treatment phases should be randomized to correct for confounders such as seasonal influences and (unexpected) learning effects. In case of group comparisons, such as in a crossover study, randomization should be stratified based on predefined criteria, in order to create comparable groups. Secondly, since all of the above‐mentioned study designs are subject to report bias, blinding of both investigators and patients is of great importance, especially in case of subjective end points. Finally, since the outcome measure is applied multiple times within the same subject, the outcome measures should be tested for learning processes, which is expected to be the case for, for example, the 6‐min walking test (Casey *et al*, 2012). Another design is the “Bayesian trial design” (Berry, 2006), which gives information about the probability that a predefined change occurs (e.g., the chance that the GMFM score is 10% higher after 4 weeks of active treatment compared to the placebo condition). Although the Bayesian design might still be unfamiliar to regulatory authorities and clinical investigators, its flexible design and smaller sample sizes make it a promising design for phase 3 trials.

### A case study: the development of KH176

Obviously, results obtained with (patient‐derived) cell lines (as any other model system including mice) should be treated with caution when translating to human pathology. As mentioned above, we hypothesized that treatment with the vitamin E‐derived antioxidant Trolox might be a viable starting point for the rational development of small molecules that correct or mitigate the cellular consequences of nDNA‐encoded complex I mutations. To allow its effective use in animal models and human subjects, we developed a first library of 20 novel Trolox variants, several of which displayed increased potency and efficacy in scavenging cellular ROS. However, small chemical molecules generally induce multiple subtle effects. This means that techniques like single‐parameter high‐throughput cell screening and *in vitro* potency analysis are not generally predictive to identify the most optimal molecules. By combining automated image quantification and machine learning techniques, we were able to discriminate between the mitochondrial morpho‐functional phenotype of fibroblasts of a healthy individual and a Leigh syndrome patient (Blanchet *et al*, 2015). Application of this strategy highlighted Trolox ornithylamide hydrochloride (KH003) as a hit compound that effectively scavenged ROS and increased the maximal activity of mitochondrial complex I, complex IV, and citrate synthase. Next, a second generation of compounds was developed to further improve the drug‐like properties and potency of KH003 by modifying its chemical structure through classical medicinal chemistry. The lead optimization was targeted and prioritized toward the improvement of solubility, cell permeability, oral bioavailability, blood–brain barrier permeability, and chemical stability. The potency and efficacy of this 2^nd^‐generation library in scavenging cellular ROS and protecting cells against oxidative stress was again analyzed in Leigh syndrome patient fibroblasts. This strategy finally yielded KH176 as a lead compound since it displayed the best compromise between potency, drug‐like properties and the *in vitro* therapeutic window (i.e., the space between potency and toxicity). Following successful regulatory toxicology analysis, KH176 has entered the clinical trial phase ( www.clinicaltrials.gov; NCT02544217) and recently successfully completed the clinical trial phase 1.

### Small molecules for treatment of mitochondrial disease

In addition to KH176, various small molecules for treatment of mitochondrial diseases are currently being evaluated in clinical studies (Kerr, 2013; Rai *et al*, 2015). These include idebenone, EPI‐743, MTP‐131, RP103, coenzyme Q_10_, bezafibrate, RG2133, DCA, ARG/CIT, lipoic acid, RTA408, and curcumin (Table 2).

**Table 2:** Small molecules used in clinical studies for treatment of mitochondrial diseases

Molecule name	Targeted disease	Clinical phase	Clinical trial identifier^a^	Primary results	Sponsor
KH176	MELAS	Phase 1 double‐blind, randomized, placebo‐controlled study of the safety, tolerability, pharmacokinetics, and pharmacodynamics in healthy volunteers	NCT02544217	KH176 is well tolerated and displays a promising PK profile	Khondrion, Nijmegen, the Netherlands
Idebenone (a.k.a. Catena®, Raxone®, Sovrima®)	LHON	Phase 2 double‐blind, randomized, placebo‐controlled study of the efficacy, safety, and tolerability	NCT00747487	The primary end point did not reach statistical significance. In a subgroup of patients with discordant visual acuities at baseline, all secondary end points were significantly different between the idebenone and placebo groups	Santhera Pharmaceuticals, Liestal, Switzerland
	FRDA	Phase 3 double‐blind, randomized, placebo‐controlled study of the efficacy, safety, and tolerability	NCT00537680	Idebenone did not significantly alter neurological function in FRDA during the 6‐month study	
	MELAS	Phase 2a double‐blind, randomized, placebo‐controlled, dose‐finding study	NCT00887562	No results reported	
EPI‐743 (a.k.a. Vatiquinone®, Vincerinone®)	LS	Phase 2B randomized, placebo‐controlled, double‐blind clinical trial	NCT01721733	Study status unknown	Edison Pharmaceuticals, Mountain View, USA
	FRDA	Safety and efficacy study on visual function	NCT01728064	Ongoing, not recruiting	
	MRCD	Emergency use protocol for EPI‐743 in acutely ill patients with inherited MRCD (90 days of end‐of‐life care)	NCT01370447	Ongoing, not recruiting	
	PS	Open‐label phase 2 safety and efficacy study	NCT02104336	Ongoing, not recruiting	
	FRDA	Phase 2A clinical trial on visual function in patients with point mutations	NCT01962363	Ongoing, not recruiting	University of South Florida, Tampa, USA
MTP‐131 (a.k.a. Bendavia®)	MM	Phase 1/2 multicenter, randomized, double‐blind, placebo‐controlled, multiple ascending‐dose clinical study investigating the safety, tolerability, and efficacy of intravenous MTP‐131 for the treatment of MM in subjects with genetically confirmed MD	NCT02367014	Recruiting	Stealth BioTherapeutics Inc., Newton, USA
	SMMDE	Phase 2 randomized, double‐blind, placebo‐controlled study to evaluate the impact of a single intravenous dose	NCT02245620	Recruiting	
RP103 (Cysteamine bitartrate)	Inherited MD including LS	A phase 2/3 open‐label, dose‐escalating study to assess safety, tolerability, efficacy, PK, and PD of RP103 delayed‐release capsules in children	NCT02023866 RP103‐MITO‐001	Recruiting	Raptor pharmaceuticals, Novato, USA
		A phase 2 long‐term open‐label extension study of RP103‐MITO‐001 to assess the safety, tolerability and efficacy of RP103 delayed‐release capsules for treatment of children	NCT02473445	Recruiting	
Coenzyme Q_10_	Children inherited MD due to defects in specific ETC complexes or mtDNA mutations	Phase 3 trial	NCT00432744	Completed. No results reported yet	University of Florida; FDA office of orphan products development, USA
Bezafibrate	MD (Confirmed mt.3243A>G mutation)	Open‐label phase 2 feasibility study	NCT02398201	Recruiting not started	Newcastle‐upon‐Tyne Hospitals NHS foundation Trust, Newcastle, UK
RG2133 (2′,3′,5′‐tri‐O‐acetyluridine)	MD	Open‐label dose‐escalation phase I study to assess the safety, tolerability, PK, and PD of RG2133 in treatment of inherited MD	NCT00060515	Terminated. No results reported	Repligen Corp, Waltham, USA
DCA (dichloroacetate)	MELAS	Phase 2: investigation of clinical syndromes associated with mtDNA point mutations	NCT00068913	Terminated prematurely because of peripheral nerve toxicity	Eunice Kennedy Shriver NICHD, Bethesda, USA
ARG and CIT	MELAS	Open‐label phase 2: ARG flux and NO production in patients and the effect of dietary ARG and CIT supplementation	NCT01339494	Unknown	Baylor College of Medicine, Houston, USA
ARG	MELAS	Open‐label phase 2 on 3 siblings: Efficacy of L‐arginine therapy on endothelium‐dependent vasodilation and mitochondrial metabolism	NCT01603446	Completed. A significant increase of the maximum work performed at anaerobic threshold was observed	The Hospital for Sick Children, Ontario, Canada
Lipoic acid (a.k.a. Thioctic acid)	MM	Pilot compassionate use study	NCT00004770	No results reported	National Center for Research Resources (NCRR), Bethesda, USA
RTA408	MM	Phase 2 study of the safety, efficacy, and PD of the Nrf2‐activator RTA408	NCT02255422	Recruiting	Reata Pharmaceuticals Inc, Irving, USA
Curcumin	LHON	Phase 3 randomized, double‐blind, placebo‐controlled trial	NCT00528151	No results reported	Mahidol University, Salaya, Thailand

a Source: www.clinicaltrials.gov .

ARG, arginine; CIT, citrulline; ETC, electron transport chain; FDA, Food and Drug Administration; FRDA, Friedreich's Ataxia; GLC, glucose; LHON, Leber hereditary optic neuropathy; LS, Leigh syndrome; MD, mitochondrial disease; MELAS, mitochondrial encephalomyopathy, lactic acidosis, and stroke‐like episodes; MM, mitochondrial myopathy; MRCD, mitochondrial respiratory chain diseases; mtDNA, mitochondrial DNA: NICHD, National Institute of Child Health and Human Development; NO, nitric oxide; Nrf2 (a.k.a. NFE2L2), nuclear factor (erythroid‐derived 2)‐like 2; PD, pharmacodynamics; PK, pharmacokinetics; PS, Pearson syndrome; SMMDE, skeletal muscle mitochondrial dysfunction in the elderly.

- Idebenone can act as an antioxidant and delivers electrons directly to complex III, thereby bypassing a deficient complex I (Giorgio *et al*, 2011; Haefeli *et al*, 2011; Jaber & Polster, 2015). This compound has recently received market approval in Europe for the treatment of LHON. It is also under clinical investigation for the treatment of MELAS (phase 2a studies) and Friedreich ataxia (phase 3 studies).
- EPI‐743 functions as a cofactor of the enzyme NAD(P)H dehydrogenase Quinone 1 (NQO1) and enhances the biosynthesis of glutathione (GSH), which is an important cellular antioxidant (Pastore *et al*, 2013). EPI‐743 is currently evaluated in clinical phase 2 studies in various indications such as Leigh, LHON, and Pearson syndrome. Promising results though from initial single open‐label trials in patients suffering from Leigh syndrome (Enns *et al*, 2012; Martinelli *et al*, 2012) or LHON syndrome (Sadun *et al*, 2012) have been reported.
- MTP‐131 is a member of the Szeto‐Schiller (SS) peptide family and binds to the mitochondria‐specific MIM lipid cardiolipin (Alam *et al*, 2015). It increases OXPHOS efficiency and potentially improves the ability of mitochondria to properly respond to metabolic changes. Using different formulations, MTP‐131 has entered clinical trials for the treatment of mitochondrial myopathies (phase 1/2) and LHON (phase 1/2). In pre‐clinical studies, MTP‐131 corrected excessive ROS generation and increased ATP production in mitochondrial dysfunction (Szeto, 2014).
- RP103 is intended to supply the cell with additional cysteine, an important amino acid for GSH neosynthesis (Besouw *et al*, 2012). RP103 is currently being evaluated in the form of delayed‐release capsules in the treatment of children with inherited mitochondrial disease, including Leigh syndrome (phase 2/3).
- Coenzyme Q_10_ is an ETC component involved in electron transport (Fig 1B), which also displays antioxidant capacity. It is available without prescription and has been evaluated in a phase 2 study on adult patients with mitochondrial cytopathies (MELAS, LHON, and chronic progressive external ophthalmoplegia; CPEO), showing minor improvement (Glover *et al*, 2010). A phase 3 study (Stacpoole *et al*, 2012) has been completed in children with primary mitochondrial diseases but, as far as we know, no results have been reported yet.
- Bezafibrate is a known activator of the transcription factor peroxisomal proliferator‐activated receptors (PPARs; Wenz *et al*, 2008). When activated, PPARs induce transcription of genes involved in mitochondrial function including fatty acid metabolism. Effects of bezafibrate are conflicting, since it both displayed adverse effects and positive effects in various studies (Wenz *et al*, 2008; Viscomi *et al*, 2011; Yatsuga & Suomalainen, 2012). The therapeutic potential of bezafibrate is currently evaluated in an open‐labeled phase 2 study on patients carrying the mtDNA 3243A>G mutation. The latter mutation is one of the most common mtDNA mutations in mitochondrial disease and is causatively linked to MELAS, CPEO, and maternally inherited deafness and diabetes (MIDD; Nesbitt & McFarland, 2011).
- RG2133 or 2′,3′,5′‐tri‐O‐acetyluridine is a precursor of uridine, a pyrimidine nucleotide involved in RNA and DNA synthesis. Endogenous uridine synthesis depends on coenzyme Q_10_ recycling and normal OXPHOS function. This means that OXPHOS dysfunction can impair uridine synthesis leading to cellular dysfunction. The assessment of RG2133 safety and efficacy in a phase 1 study on patients with mitochondrial disease was terminated. As far as we know, no results have been reported.
- DCA (dichloroacetate) is a structural analog of pyruvate. This molecule locks PDHC (which converts pyruvate into acetyl‐coA), in an active state by inhibiting pyruvate dehydrogenase kinase. This might improve OXPHOS function despite the presence of PDHC or OXPHOS defects. A randomized controlled phase 2 trial in patient suffering from MELAS was terminated prematurely because of peripheral nerve toxicity (Kaufmann *et al*, 2006).
- ARG/CIT (L‐arginine/L‐citrulline) are precursors of nitric oxide (NO), an important cellular‐signaling molecule involved in vasodilation. Therefore, increased NO production following supplementation with ARG/CIT is hypothesized to protect MELAS patients against recurrent strokes possibly due to vasoconstriction. Open‐label studies in MELAS patients have shown promising results (Rodan *et al*, 2015), but still need be performed under well‐controlled conditions to draw final conclusions.
- Lipoic acid displays antioxidant activity *in vivo* (Koufaki, 2014). This compound was evaluated in a combination treatment with creatine and coenzyme Q_10_ during a phase 2 randomized and controlled trial on patients with mitochondrial cytopathies (Rodriguez *et al*, 2007). During this trial, positive effects on surrogate markers of oxidative stress were observed. It was also studied alone in a compassionate use program using a single patient with mitochondrial myopathy, but no results are currently available.
- RTA408 activates the cytoprotective transcription factor nuclear factor (erythroid‐derived 2)‐like 2 (Nrf2, a.k.a. NFE2L2), a master regulator of cellular antioxidant responses (Reisman *et al*, 2014). This compound has entered clinical trials for the treatment of mitochondrial myopathy (phase 2) and is reported to stimulate mitochondrial biogenesis and function (Neymotin *et al*, 2011), and increase the expression of cellular antioxidant defense systems (Saha *et al*, 2010).
- Curcumin, a biomolecule from the golden spice turmeric (*Curcuma longa*), can modulate multiple cell‐signaling pathways and displays antioxidant properties (Gupta *et al*, 2011; Trujilo *et al*, 2014). A randomized and controlled phase 3 study in LHON patients carrying the G11778A mtDNA, mutation has been completed but no results have been reported yet.

Various other small‐molecule treatments are currently under investigation but still at the early preclinical stage. For example, a novel cell‐permeable variant of the complex II substrate succinate (NVP015; Neurovive Pharmaceuticals, Lund, Sweden) has been generated to allow bypassing of dysfunctional complex I. In addition, using a mouse model of isolated complex I deficiency (*NDUFS4* ^−/−^ mice; Kruse *et al*, 2008), positive effects of the mTOR inhibitor rapamycin (Johnson *et al*, 2013, 2015), nicotinamide riboside (a natural precursor of NAD^+^) (Karamanlidis *et al*, 2013), and inhibition of poly ADP ribose polymerase (PARP) have been reported (Felici *et al*, 2014). Both nicotinamide riboside and PARP inhibition are thought to improve mitochondrial function by enhancing intracellular NAD^+^ levels (Cerutti *et al*, 2014). Further potential strategies, including mitochondria‐targeted small molecules, have recently been presented elsewhere (e.g., Horobin *et al*, 2007; Karamanlidis *et al*, 2013; Apostolova & Victor, 2014; Khan *et al*, 2014; Malti *et al*, 2014; Peralta *et al*, 2015; Rai *et al*, 2015; Viscomi *et al*, 2015).

## Future perspectives

There is an unmet need for development of clinically relevant treatment strategies for children (and adults) suffering from mitochondrial disease. Developing such a treatment faces many challenges including the small number of established diagnostic and prognostic biomarkers, the lack of truly objective and validated outcome measures that reflect daily life activities/well‐being, and the small number of homogenous patients. To systematically overcome these hurdles, (more) international collaboration between mitochondrial expert centers, patient organizations, industrial partners, and other stakeholders, as well as sufficient funding options, is essential. The tasks ahead include the establishment of guidelines for high‐quality studies, and to improve trial design, for instance, by harmonization of outcome measures.

## Conflict of interest

JAMS is the founding CEO of Khondrion ( www.khondrion.com), a Radboud University Medical Center spin‐off biotech company. JB is the COO, and PHGMW and WJHK are scientific consultants of this company.

**Pending issues**

- *Outcome measures*: The validation and harmonization of objective clinically relevant outcome measures in children with mitochondrial disease is a crucial step in the interpretation of drug effectiveness. This requires an active stimulation of patient participation and warrants further international collaboration. The same holds true for diagnostic biomarkers suited for use during intervention studies and prognosis.
- *Natural history studies*: Due to the relatively limited number of pediatric patients in which a genetic defect is firmly established, international initiatives are required for proper intervention testing.
- *Clinical trials*: Although many different designs of clinical trials exist, their results should be carefully interpreted and include clinical relevant primary endpoints. The latter are dictated by the available population of affected patients.
- *Genotype–phenotype relationship*: It is unclear why mutations in different genes are associated with similar clinical phenotypes and why similar mutations are associated with different phenotypes. Moreover, it is unknown why certain mutations display tissue‐specific effects. Environmental and/or patient‐specific factors might explain such differences and therefore require further exploration.
